# Research impact: a narrative review

**DOI:** 10.1186/s12916-016-0620-8

**Published:** 2016-05-23

**Authors:** Trisha Greenhalgh, James Raftery, Steve Hanney, Matthew Glover

**Affiliations:** Nuffield Department of Primary Care Health Sciences, University of Oxford, Radcliffe Primary Care Building, Woodstock Rd, Oxford, OX2 6GG UK; Primary Care and Population Sciences, Faculty of Medicine, University of Southampton, Southampton General Hospital, Southampton, SO16 6YD UK; Health Economics Research Group (HERG), Institute of Environment, Health and Societies, Brunel University London, ᅟ, UB8 3PH UK

**Keywords:** Research impact, Knowledge translation, Implementation science, Research utilization, Payback Framework, Monetisation, Research accountability, Health gains

## Abstract

Impact occurs when research generates benefits (health, economic, cultural) in addition to building the academic knowledge base. Its mechanisms are complex and reflect the multiple ways in which knowledge is generated and utilised. Much progress has been made in measuring both the outcomes of research and the processes and activities through which these are achieved, though the measurement of impact is not without its critics. We review the strengths and limitations of six established approaches (Payback, Research Impact Framework, Canadian Academy of Health Sciences, monetisation, societal impact assessment, UK Research Excellence Framework) plus recently developed and largely untested ones (including metrics and electronic databases). We conclude that (1) different approaches to impact assessment are appropriate in different circumstances; (2) the most robust and sophisticated approaches are labour-intensive and not always feasible or affordable; (3) whilst most metrics tend to capture direct and proximate impacts, more indirect and diffuse elements of the research-impact link can and should be measured; and (4) research on research impact is a rapidly developing field with new methodologies on the horizon.

## Background

This paper addresses the question: ‘What is research impact and how might we measure it?’. It has two main aims, first, to introduce the general reader to a new and somewhat specialised literature on the science of research impact assessment and, second, to contribute to the development of theory and the taxonomy of method in this complex and rapidly growing field of inquiry. Summarising evidence from previous systematic and narrative reviews [1–7], including new reviews from our own team [1, 5], we consider definitions of impact and its conceptual and philosophical basis before reviewing the strengths and limitations of different approaches to its assessment. We conclude by suggesting where future research on research impact might be directed.

Research impact has many definitions (Box 1). Its measurement is important considering that researchers are increasingly expected to be accountable and produce value for money, especially when their work is funded from the public purse [8]. Further, funders seek to demonstrate the benefits from their research spending [9] and there is pressure to reduce waste in research [10]. By highlighting how (and how effectively) resources are being used, impact assessment can inform strategic planning by both funding bodies and research institutions [1, 11].

We draw in particular on a recent meta-synthesis of studies of research impact funded by the UK Health Technology Assessment Programme (HTA review) covering literature mainly published between 2005 and 2014 [1]. The HTA review was based on a systematic search of eight databases (including grey literature) plus hand searching and reference checking, and identified over 20 different impact models and frameworks and 110 studies describing their empirical applications (as single or multiple case studies), although only a handful had proven robust and flexible across a range of examples. The material presented in this summary paper, based on much more extensive work, is inevitably somewhat eclectic. Four of the six approaches we selected as ‘established’ were the ones most widely used in the 110 published empirical studies. Additionally, we included the Societal Impact Assessment despite it being less widely used since it has recently been the subject of a major EU-funded workstream (across a range of fields) and the UK Research Excellence Framework (REF; on which empirical work post-dated our review) because of the size and uniqueness of the dataset and its significant (?) international interest. The approaches we selected as showing promise for the future were chosen more subjectively on the grounds that there is currently considerable academic and/or policy interest in them.

Different approaches to assessing research impact make different assumptions about the nature of research knowledge, the purpose of research, the definition of research quality, the role of values in research and its implementation, the mechanisms by which impact is achieved, and the implications for how impact is measured (Table 1). Short-term proximate impacts are easier to attribute, but benefits from complementary assets (such as the development of research infrastructure, political support or key partnerships [8]) may accumulate in the longer term but are more difficult – and sometimes impossible – to fully capture.

**Table 1:** Philosophical assumptions underpinning approaches to research impact

Perspective	Positivist	Constructivist	Realist	Critical	Performative
Assumptions about what [research] knowledge is	Facts (especially statements on relationships between variables), independent of researchers and transferable to new contexts	Explanations/interpretations of a situation or phenomenon, considering the historical, cultural and social context	Studies of how people interpret external reality, producing statements on ‘what works for whom in what circumstances’	Studies that reveal society’s inherent conflicts and injustices and give people the tools to challenge their oppression	Knowledge is brought into being and enacted in practice by actor-networks of people and technologies
Assumed purpose of research	Predictive generalisations (‘laws’)	Meaning: perhaps in a single, unique case	Theoretical generalisation (what tends to work and why)	Learning, emancipation, challenge	To map the changing dynamics of actor-networks
Preferred research methods	Hypothesis-testing; experiments; modelling and measurement	Naturalistic inquiry (i.e. in real-world conditions)	Predominantly naturalistic, may combine quantitative and qualitative data	Participatory [action] research	Naturalistic, with a focus on change over time and network [in]stability
Assumed way to achieve quality in research	Hierarchy of preferred study designs; standardised instruments to help eliminate bias	Reflexive theorising; consideration of multiple interpretations; dialogue and debate	Abduction (what kind of reasoning by human actors could explain these findings in this context?)	Measures to address power imbalances (ethos of democracy, conflict management); research capacity building in community partner(s)	Richness of description; plausible account of the network and how it changes over time
Assumed relationship between science and values	Science is inherently value-neutral (though research can be used for benign or malevolent motives)	Science can never be value-neutral; the researcher’s perspective must be made explicit	Facts are interpreted and used by people who bring particular values and views	Science must be understood in terms of what gave rise to it and the interests it serves	Controversial; arguably, Actor-Network Theory is consistent with a value-laden view of science
Assumed mechanism through which impact is achieved	Direct (new knowledge will influence practice and policy if the principles and methods of implementation science are followed)	Mainly indirect (e.g. via interaction/enlightenment of policymakers and influencing the ‘mindlines’ of clinicians)	Interaction between reasoning (of policymakers, practitioners, etc.) and resources available for implementing findings	Development of critical consciousness; partnership-building; lobbying; advocacy	‘Translations’ (stable changes in the actor-network), achieved by actors who mobilise other actors into new configurations
Implications for the study of research impact	‘Logic models’ will track how research findings (transferable facts about what works) are disseminated, taken up and used for societal benefit	Outcomes of social interventions are unpredictable; impact studies should focus on ‘activities and interactions’ to build relations with policymakers	Impact studies should address variability in uptake and use of research by exploring context-mechanism-outcome-impact configurations	Impact has a political dimension; research may challenge the status quo; some stakeholders stand to lose power, whereas others may gain	For research to have impact, a re-alignment of actors (human/technological) is needed; focus on the changing ‘actor-scenario’ and how this gets stabilised in the network

Knowledge is intertwined with politics and persuasion. If stakeholders agree on what the problem is and what a solution would look like, the research-impact link will tend to turn on the strength of research evidence in favour of each potential decision option, as depicted in column 2 of Table 1 [12]. However, in many fields – for example, public policymaking, social sciences, applied public health and the study of how knowledge is distributed and negotiated in multi-stakeholder collaborations – the links between research and impact are complex, indirect and hard to attribute (for an example, see Kogan and Henkel’s rich ethnographic study of the Rothschild experiment in the 1970s, which sought – and failed – to rationalize the links between research and policy [13]). In policymaking, research evidence is rather more often used conceptually (for general enlightenment) or symbolically (to justify a chosen course of action) than instrumentally (feeding directly into a particular policy decision) [12, 14], as shown empirically by Amara et al.’s large quantitative survey of how US government agencies drew on university research [15]. Social science research is more likely to illuminate the complexity of a phenomenon than produce a simple, ‘implementable’ solution that can be driven into practice by incorporation into a guideline or protocol [16, 17], as was shown by Dopson and Fitzgerald’s detailed ethnographic case studies of the implementation of evidence-based healthcare in healthcare organisations [18]. In such situations, the research-impact relationship may be productively explored using approaches that emphasise the fluidity of knowledge and the multiple ways in which it may be generated, assigned more or less credibility and value, and utilised (columns 3 to 6 in Table 1) [12, 19].

Many approaches to assessing research impact combine a logic model (to depict input-activities-output-impact links) with a ‘case study’ description to capture the often complex processes and interactions through which knowledge is produced (perhaps collaboratively and/or with end-user input to study design), interpreted and shared (for example, through engagement activities, audience targeting and the use of champions, boundary spanners and knowledge brokers [20–24]). A nuanced narrative may be essential to depict the non-linear links between upstream research and distal outcomes and/or help explain why research findings were not taken up and implemented despite investment in knowledge translation efforts [4, 6].

Below, we describe six approaches that have proved robust and useful for measuring research impact and some additional ones introduced more recently. Table 2 lists examples of applications of the main approaches reviewed in this paper.

**Table 2:** Examples of applications of research impact assessment frameworks

Author/year (country)	Approach taken	Main findings	Comment
Payback Framework			
Kwan et al., 2007 [67] (Hong Kong)	Surveyed 205 projects funded by the Health and Health Services Research fund; used main Payback categories and framework processes	Between a third and a half of principal investigators claimed impact on policy, practice and health service benefit; liaison with potential users and participation in policy committees was significantly associated with achieving wider impacts	Multivariate analysis of data enabled identification of factors associated with impact; however, study relied solely on self-reported data from researchers
Hanney et al., 2007 [7] (UK)	16 case studies randomly selected from wider survey of all projects funded by the NHS Health Technology Assessment (HTA) programme 1993–2003; survey data supplemented by documentary and bibliometric analysis and researcher interviews	Survey showed considerable impact in knowledge production (publications), changes in policy (73 % of projects) and behaviour (42 %); case studies showed diversity in levels and forms of impacts and ways in which they arose; studies commissioned for policy customers showed highest policy impact	All case studies were written up around stages of Payback, which facilitated cross-case analysis; affirmed the value of agenda setting to meet needs of healthcare system
Scott et al., 2011 [68] (USA) (methods) and Madrillon Group, 2011 [69] (findings)	Assessed impact of National Institutes of Health’s (NIH) Mind Body Interactions and Health programme; for centres and projects: documentary review, bibliometric and database analysis, interviews; impact of centres scored using Payback scales	Findings covered programme as a whole, centres, and research projects; study demonstrated that centres and projects had produced clear and positive impacts across all five Payback categories; for projects, 34 % claimed impact on policies, 48 % led to improved health	Payback was adaptable to meet needs of specific evaluation, covering different levels; assessment occurred too early to capture many of the ‘latent’ outcomes
Hanney et al., 2013 [70] (UK)	Assessed impact of Asthma UK’s portfolio of funding including projects, fellowships, professorial chairs and a new collaborative centre; surveys to 163 researchers, interviews, documentary analysis, 14 purposively selected case studies	Findings highlighted academic publications, and considerable leverage of follow-on funding; each of the wider impacts (informing guidelines, product development, improved health) achieved by only a small number of projects or fellowships – but some significant examples, especially from chairs	The charity used the findings to inform their research strategy, notably in relation to centres; many impacts were felt to be at an early stage
Donovan et al., 2014 [71] (Australia)	Assessed impact of research funded by National Breast Cancer Foundation; survey of 242 researchers, document analysis plus 16 purposively selected case studies; considered basic and applied research and infrastructure; cross-case analysis	Impacts included academic publications, research training, research capacity building, leveraged additional funding, changed policy (10 %, though 29 % expected to do so), new product development (11 %), changed clinical practice (14 %)	The charity considered that findings would help to inform their research strategy; many projects recently completed, hence emphasis on expected impacts
Wooding et al., 2014 [72] (Australia, Canada, UK)	29 case studies randomly selected from cardiovascular/stroke research funders, scored using Payback categories; compared impact scores with features of research processes	Wide range of impacts; some projects scored very high, others very low; basic research had higher academic impacts, clinical had more impact beyond academia; engagement with practitioners/patients linked to academic and wider impacts	Payback enabled collection of data about a wide range of impacts plus processes/features of each project; this facilitated innovative analysis of factors associated with impact
Research Impact Framework			
Kuruvilla et al., 2007 [32] (UK)	Pilot study, 11 projects; used semi-structured interview and document analysis, leading to one-page ‘researcher narrative’ that was sent to the researcher for validation	Interviews with researchers allowed them to articulate and make sense of multiple impact channels and activities; the structured researcher narratives, which were objectively verifiable, facilitated comparison across projects	Applied a wider range of impact categories than the Payback Framework; approach was adaptable and acceptable to researchers, however, it was only a small pilot conducted in the researchers’ group
Canadian Academy of Health Sciences (CAHS) Framework			
Montague and Valentim, 2010 [73] (Canada)	Applied the CAHS Framework to assess the impact of a large randomised trial of a new treatment for breast cancer; divided the impacts into proximate (e.g. changes in awareness) and more long-term (including changes in breast cancer mortality)	Numerous impacts were documented at different levels of the CAHS Framework; findings suggested a direct link between publication of the trial, change in clinical practice and subsequent reduction in morbidity and mortality	Published as an early worked example of how CAHS can inform the systematic documentation of impacts
Adam et al., 2012 [74] (Catalonia)	Applied the CAHS Framework to assess the impact of clinical and health services research funded by the main Catalan agency; included bibiliometric analysis, surveys to 99 researchers with 70 responses, interviews with researchers and decision-makers, in-depth case study of translation pathways, as well as a focus on intended impacts	In the CAHS category of informing decision-making by policymakers, managers, professionals, patients, etc. 40 out of 70 claimed decision-making changes were induced by research results: 29 said changed clinical practice, 16 said organisational/policy changes; interactions in projects with healthcare and policy decision-makers was crucial	The study provided both knowledge to inform the funding agency’s subsequent actions and a basis on which to advocate for targeted research to fill knowledge gaps; the team noted limitations in relation to attribution, time lags and the counterfactual
Graham et al., 2012 [75] (Canada)	Adapted and applied CAHS to assess impact of research funded by a not-for-profit research and innovation organization in Alberta, Canada	After a formal adaptation phase, CAHS proved flexible and robust both retrospectively (to map pre-existing data) and prospectively (to track new programmes); some new categories were added	Had a particular focus on developing data capture approaches for the many indicators identified; also a focus on how the research funding organisation could measure its own contribution to achieving health system impacts
Cohen et al., 2015 [76] (Australia)	Adapted categories from Payback and CAHS; mixed method sequential methodology; surveys and interviews of lead researchers (final sample of 50); data from surveys, interviews and documents collated into case studies which were scored by an expert panel using criteria from the UK Research Excellence Framework (REF)	19 of 50 cases had policy and practice impacts with an even distribution of high, medium and low impact scores across the (REF-based) criteria of corroboration, attribution, reach and importance; showed that real world impacts can occur from single intervention studies	Innovative approach by blending existing frameworks; limitations included not always being able to obtain documentary evidence to corroborate researcher accounts
Monetisation Models			
Johnston et al., 2006 [34] (USA)	Collated data on 28 Phase III clinical trials funded by the National Institute of Neurological Disorders and Stroke up to 2000; compared monetised health gains achieved by use of new healthcare interventions (measured in QALYs and valued at GDP per head) to investment in research, using cost-utility analyses and actual usage	$335 m research investment generated 470,000 QALYs 10 years post funding; return on investment was 46 % per year	Used a bottom-up approach to quantify health gains through individual healthcare interventions; assumed that all changes in usage were prompted by NIH phase III trials; no explicit time-lag; highlights data difficulties in bottom-up approach, as required data were only available for eight trials
Access Economics, 2008 [39] (Australia)	Quantified returns from all Australian health R&D funding between 1992/3 and 2004/5. Monetised health gains estimated as predicted DALYs averted in 2033–45 compared to 1993 (valued at willingness to pay for a statistical life-year)	Return on investment of 110 % from private and public R&D; assumed that 50 % of health gains are attributable to R&D, of which 3.04 % is Australian R&D	Top-down approach; high uncertainty and sensitivity of results in 50 % assumption; forecasted future health gains
Buxton et al., 2008 [38] (UK)	Estimated returns from UK public and charitably funded cardiovascular research 1975–1988; data from cost-utility studies and individual intervention usage; health gains expressed as monetised QALYs (valued at healthcare service opportunity cost) net costs of delivery for years 1986–2005	Internal rate of return of 9 % a year, plus a component added for non-health economic ‘spill-over’ effects of 30 %; assumed a 17 year lag between investment and health gains (based on guideline analysis – knowledge cycle time), and 17 % of health gains attributable to UK research	Bottom-up approach; judgement on which interventions to include was required; explicit investigation of time-lag
Deloitte Access Economics, 2011 [35] (Australia)	Applied same methods as Access Economics (2008); quantified returns from National Health and Medical Research Council funding 2000–2010, focusing on five burdensome disease areas; monetised health gains estimated as predicted DALYs averted in 2040–50 compared to 2000, valued at willingness to pay for a statistical life-year	Return on investment ranged from 509 % in cardiovascular disease to –30 % for muscular dystrophy research; assumed that 50 % of health gains are attributable to R&D, of which 3.14 % was Australian R&D and 35 % of that is NHMRC; assumed time lag of 40 years between investment and benefit	Top-down approach; added layer in attribution problem (because it was a programme rather than totality of research funding)
Societal Impact Assessment and Related Approaches			
Spaapen et al., 2007 [46] (Netherlands)	Mainly a methodological report on the Sci-Quest Framework with brief case examples including one in pharmaceutical sciences; proposed mixed-method case studies using qualitative methods, a quantitative instrument called contextual response analysis and quantitative assessment of financial interactions (grants, spin-outs, etc.). Produced a bespoke Research Embedment and Performance Profile (REPP) for each project	Productive interactions (direct, indirect, financial) must happen for impact to occur; there are three social domains: science/certified knowledge, industry/market and policy/societal; REPP in pharmaceutical sciences example developed 15 benchmarks (five for each domain) and scored on 5-point scale	Illustrates ‘performative’ approach to impact (column 6 in Table 1); ERiC (Evaluating Research in Context) programme, focuses assessment on the context and is designed to overcome what were seen as the linear and deterministic assumptions of logic models, but complex to apply
Molas-Gallart and Tang, 2011 [77] (UK)	Applied SIAMPI Framework to assess how social science research in a Welsh university supports local businesses; case study approach using two structured questionnaires – one for researchers and one for stakeholders	Authors found few, if any, examples of linear research-impact links but “*a mesh of formal and informal collaborations in which academics are providing support for the development of specific business models in emerging areas, many of which have not yet yielded identifiable impacts*”	Good example from outside the medical field of how SIAMPI Framework can map the processes of interaction between researchers and stakeholders
UK Research Excellence Framework (secondary analyses of REF impact case study database)			
Hinrichs and Grant, 2015 [78] (UK)	Preliminary analysis of all 6679 non-redacted impact case studies in REF 2014, based mainly but not exclusively on automated text mining	Text mining identified 60 different kinds of impact and 3709 ‘pathways to impact’ through which these had (according to the authors) been achieved; researchers’ efforts to monetise health gains (e.g. as QALYs) appeared crude and speculative, though in some cases the evaluation team were able (with additional efforts) to produce monetised estimates of return on investment	Authors commented: “*the information presented in the* [REF impact] *case studies was neither consistent nor standardised.*” There is potential to improve data collection and reporting process for future exercises
Greenhalgh and Fahy, 2015 [79] (UK)	Manual content analysis of all 162 impact case studies submitted to a single sub-panel of the REF, with detailed interpretive analysis of four examples of good practice	REF impact case study format appeared broadly fit for purpose but most case studies described ‘surrogate’ and readily verifiable impacts, e.g. changing a guideline; models of good practice were characterised by proactive links with research users	Sample was drawn from a single sub-panel (public health/health services research), so findings may not be generalizable to other branches of medicine
Realist Evaluation			
Rycroft-Malone et al., 2015 [56] (UK)	In the national evaluation of first-wave Collaborations for Leadership in Applied Health Research and Care (CLAHRCs), qualitative methods (chiefly, a series of stakeholder interviews undertaken as the studies unfolded) were used to tease out actors’ theories of change and explore how context shaped and constrained their efforts to both generate and apply research knowledge	Impact in the applied setting of CLAHRCs requires commitment to the principle of collaborative knowledge production, facilitative leadership and acknowledgement by all parties that knowledge comes in different forms; impacts are contingent and appear to depend heavily on how different partners view the co-production task	Illustrates realist model of research impact (column 4 in Table 1); the new framework developed for this high-profile national evaluation (Fig. 3) has yet to be applied in a new context
Participatory Research Impact Model			
Cacari-Stone et al., 2014 [60] (USA)	In-depth case study of policy-oriented participatory action research in a deprived US industrial town to reduce environmental pollution; mixed methods including individual interviews, focus groups, policymaker phone interviews, archival media and document review, and participant observation	Policy change occurred and was attributed to strong, trusting pre-existing community-campus relationships; dedicated funding for the participatory activity; respect for ‘street science’ as well as academic research; creative and effective use of these data in civic engagement activities; diverse and effective networking with inter-sectoral partners including advocacy organisations	Illustrates ‘critical’ model of research impact (column 5 in Table 1)

## Established approaches to measuring research impact

### The Payback Framework

**Fig. 1:**
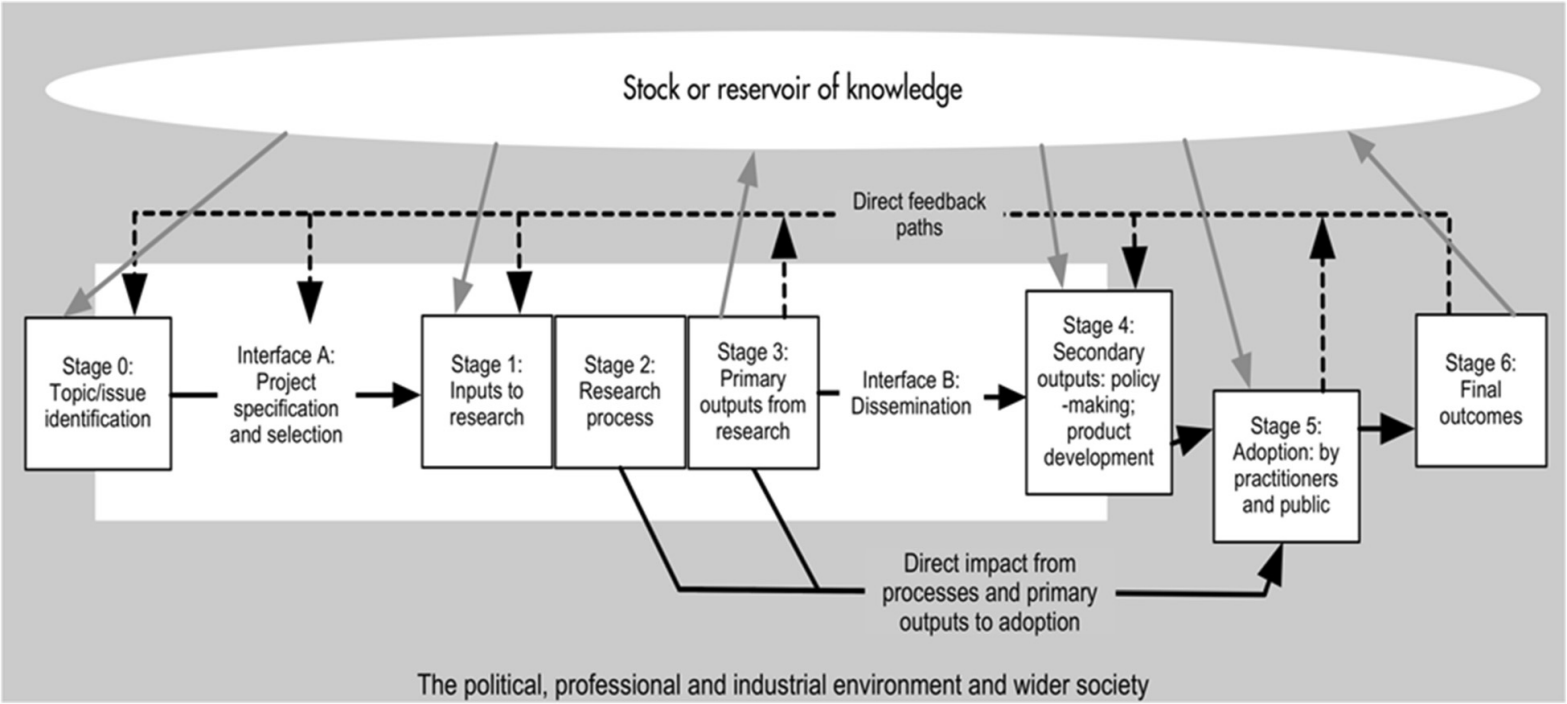
The Payback Framework developed by Buxton and Hanney (reproduced under Creative Commons Licence from Hanney et al [70])

Developed by Buxton and Hanney in 1996 [25], the Payback Framework (Fig. 1) remains the most widely used approach. It was used by 27 of the 110 empirical application studies in the recent HTA review [1]. Despite its name, it does not measure impact in monetary terms. It consists of two elements: a logic model of the seven stages of research from conceptualisation to impact, and five categories to classify the paybacks – knowledge (e.g. academic publications), benefits to future research (e.g. training new researchers), benefits to policy (e.g. information base for clinical policies), benefits to health and the health system (including cost savings and greater equity), and broader economic benefits (e.g. commercial spin-outs). Two interfaces for interaction between researchers and potential users of research (‘project specification, selection and commissioning’ and ‘dissemination’) and various feedback loops connecting the stages are seen as crucial.

The elements and categories in the Payback Framework were designed to capture the diverse ways in which impact may arise, notably the bidirectional interactions between researchers and users at all stages in the research process from agenda setting to dissemination and implementation. The Payback Framework encourages an assessment of the knowledge base at the time a piece of research is commissioned – data that might help with issues of attribution (did research A cause impact B?) and/or reveal a counterfactual (what other work was occurring in the relevant field at the time?).

Applying the Payback Framework through case studies is labour intensive: researcher interviews are combined with document analysis and verification of claimed impacts to prepare a detailed case study containing both qualitative and quantitative information. Not all research groups or funders will be sufficiently well resourced to produce this level of detail for every project – nor is it always necessary to do so. Some authors have adapted the Payback Framework methodology to reduce the workload of impact assessment (for example, a recent European Commission evaluation populated the categories mainly by analysis of published documents [26]); nevertheless, it is not known how or to what extent such changes would compromise the data. Impacts may be short or long term [27], so (as with any approach) the time window covered by data collection will be critical.

Another potential limitation of the Payback Framework is that it is generally project-focused (commencing with a particular funded study) and is therefore less able to explore the impact of the sum total of activities of a research group that attracted funding from a number of sources. As Meagher et al. concluded in their study of ESRC-funded responsive mode psychology projects, “*In most cases it was extremely difficult to attribute with certainty a particular impact to a particular project’s research findings. It was often more feasible to attach an impact to a particular researcher’s full body of research, as it seemed to be the depth and credibility of an ongoing body of research that registered with users*” [28] (p. 170).

Similarly, the impact of programmes of research may be greater than the sum of their parts due to economic and intellectual synergies, and therefore project-focused impact models may systematically underestimate impact. Application of the Payback Framework may include supplementary approaches such as targeted stakeholder interviews to fully capture the synergies of programme-level funding [29, 30].

### Research Impact Framework

The Research Impact Framework was the second most widely used approach in the HTA review of impact assessment, accounting for seven out of 110 applications [1], but in these studies it was mostly used in combination with other frameworks (especially Payback) rather than as a stand-alone approach. It was originally developed by and for academics who were interested in measuring and monitoring the impact of their own research. As such, it is a ‘light touch’ checklist intended for use by individual researchers who seek to identify and select impacts from their work “*without requiring specialist skill in the field of research impact assessment*” [31] (p. 136). The checklist, designed to prompt reflection and discussion, includes research-related impacts, policy and practice impacts, service (including health) impacts, and an additional ‘societal impact’ category with seven sub-categories. In a pilot study, its authors found that participating researchers engaged readily with the Research Impact Framework and were able to use it to identify and reflect on different kinds of impact from their research [31, 32]. Because of its (intentional) trade-off between comprehensiveness and practicality, it generally produces a less thorough assessment than the Payback Framework and was not designed to be used in formal impact assessment studies by third parties.

### Canadian Academy of Health Sciences (CAHS) Framework

**Fig. 2:**
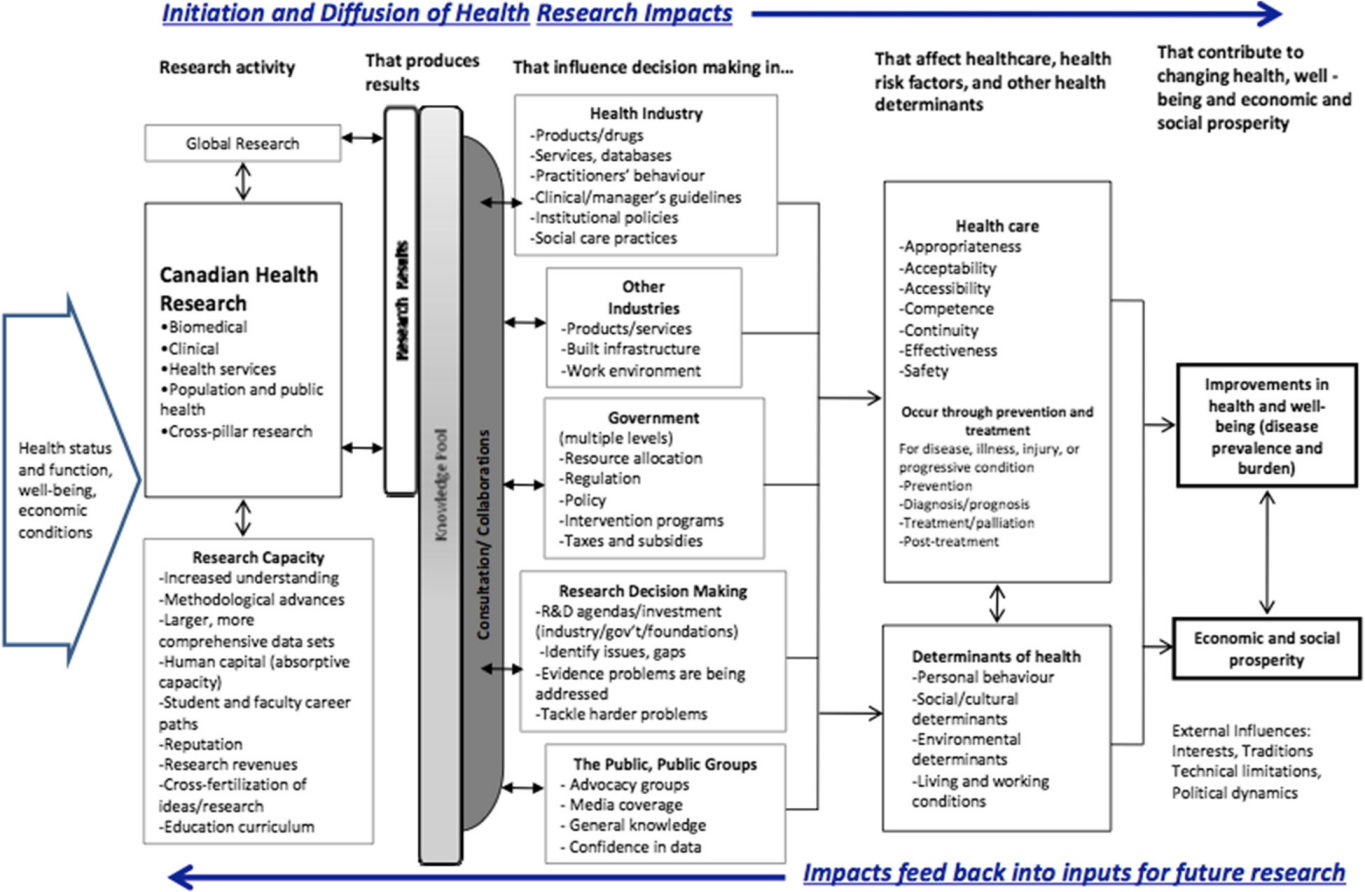
Simplified Canadian Academy of Health Sciences (CAHS) Framework (reproduced with permission of Canadian Academy of Health Sciences [33])

The most widely used adaptation of the Payback Framework is the CAHS Framework (Fig. 2), which informed six of the 110 application studies in the HTA review [33]. Its architects claim to have shaped the Payback Framework into a ‘systems approach’ that takes greater account of the various non-linear influences at play in contemporary health research systems. CAHS was constructed collaboratively by a panel of international experts (academics, policymakers, university heads), endorsed by 28 stakeholder bodies across Canada (including research funders, policymakers, professional organisations and government) and refined through public consultation [33]. The authors emphasise that the consensus-building process that generated the model was as important as the model itself.

CAHS encourages a careful assessment of context and the subsequent consideration of impacts under five categories: advancing knowledge (measures of research quality, activity, outreach and structure), capacity-building (developing researchers and research infrastructure), informing decision-making (decisions about health and healthcare, including public health and social care, decisions about future research investment, and decisions by public and citizens), health impacts (including health status, determinants of health – including individual risk factors and environmental and social determinants – and health system changes), and economic and social benefits (including commercialization, cultural outcomes, socioeconomic implications and public understanding of science).

For each category, a menu of metrics and measures (66 in total) is offered, and users are encouraged to draw on these flexibly to suit their circumstances. By choosing appropriate sets of indicators, CAHS can be used to track impacts within any of the four ‘pillars’ of health research (basic biomedical, applied clinical, health services and systems, and population health – or within domains that cut across these pillars) and at various levels (individual, institutional, regional, national or international).

Despite their differences, Payback and CAHS have much in common, especially in how they define impact and their proposed categories for assessing it. Whilst CAHS appears broader in scope and emphasises ‘complex system’ elements, both frameworks are designed as a pragmatic and flexible adaptation of the research-into-practice logic model. One key difference is that CAHS’ category ‘decision-making’ incorporates both policy-level decisions and the behaviour of individual clinicians, whereas Payback collects data separately on individual clinical decisions on the grounds that, if they are measurable, decisions by clinicians to change behaviour feed indirectly into the improved health category.

As with Payback (but perhaps even more so, since CAHS is in many ways more comprehensive), the application of CAHS is a complex and specialist task that is likely to be highly labour-intensive and hence prohibitively expensive in some circumstances.

### Monetisation models

A significant innovation in recent years has been the development of logic models to monetise (that is, express in terms of currency) both the health and the non-health returns from research. Of the 110 empirical applications of impact assessment approaches in our HTA review, six used monetization. Such models tend to operate at a much higher level of aggregation than Payback or CAHS – typically seeking to track all the outputs of a research council [34, 35], national research into a broad disease area (e.g. cardiovascular disease, cancer) [36–38], or even an entire national medical research budget [39].

Monetisation models express returns in various ways, including as cost savings, the money value of net health gains via cost per quality-adjusted life year (QALY) using the willingness-to-pay or opportunity cost established by NICE or similar bodies [40], and internal rates of return (return on investment as an annual percentage yield). These models draw largely from the economic evaluation literature and differ principally in terms of which costs and benefits (health and non-health) they include and in the valuation of seemingly non-monetary components of the estimation. A national research call, for example, may fund several programmes of work in different universities and industry partnerships, subsequently producing net health gains (monetised as the value of QALYs or disability-adjusted life-years), cost savings to the health service (and to patients), commercialisation (patents, spin-outs, intellectual property), leveraging of research funds from other sources, and so on.

A major challenge in monetisation studies is that, in order to produce a quantitative measure of economic impact or rate of return, a number of simplifying assumptions must be made, especially in relation to the appropriate time lag between research and impact and what proportion of a particular benefit should be attributed to the funded research programme as opposed to all the other factors involved (e.g. social trends, emergence of new interventions, other research programmes occurring in parallel). Methods are being developed to address some of these issues [27]; however, whilst the estimates produced in monetised models are quantitative, those figures depend on subjective, qualitative judgements.

A key debate in the literature on monetisation of research impact addresses the level of aggregation. First applied to major research budgets in a ‘top-down’ or macro approach [39], whereby total health gains are apportioned to a particular research investment, the principles of monetisation are increasingly being used in a ‘bottom-up’ [34, 36–38] manner to collect data on specific project or programme research outputs. The benefits of new treatments and their usage in clinical practice can be built up to estimate returns from a body of research. By including only research-driven interventions and using cost-effectiveness or cost-utility data to estimate incremental benefits, this method goes some way to dealing with the issue of attribution. Some impact assessment models combine a monetisation component alongside an assessment of processes and/or non-monetised impacts, such as environmental impacts and an expanded knowledge base [41].

### Societal impact assessment

Societal impact assessment, used in social sciences and public health, emphasises impacts beyond health and is built on constructivist and performative philosophical assumptions (columns 3 and 6 in Table 1). Some form of societal impact assessment was used in three of the 110 empirical studies identified in our HTA review. Its protagonists distinguish the social relevance of knowledge from its monetised impacts, arguing that the intrinsic value of knowledge may be less significant than the varied and changing social configurations that enable its production, transformation and use [42].

An early approach to measuring societal impact was developed by Spaapen and Sylvain in the early 1990s [43], and subsequently refined by the Royal Netherlands Academy of Arts and Science [44]. An important component is self-evaluation by a research team of the relationships, interactions and interdependencies that link it to other elements of the research ecosystem (e.g. nature and strength of links with clinicians, policymakers and industry), as well as external peer review of these links. Spaapen et al. subsequently conducted a research programme, Evaluating Research in Context (ERiC) [45], which produced the Sci-Quest model [46]. Later, they collaborated with researchers (who had led a major UK ESRC-funded study on societal impact [47]) to produce the EU-funded SIAMPI (Social Impact Assessment Methods through the study of Productive Interactions) Framework [48].

Sci-Quest was described by its authors as a ‘fourth-generation’ approach to impact assessment – the previous three generations having been characterised, respectively, by measurement (e.g. an unenhanced logic model), description (e.g. the narrative accompanying a logic model) and judgement (e.g. an assessment of whether the impact was socially useful or not). Fourth-generation impact assessment, they suggest, is fundamentally a social, political and value-oriented activity and involves reflexivity on the part of researchers to identify and evaluate their own research goals and key relationships [46].

Sci-Quest methodology requires a detailed assessment of the research programme in context and the development of bespoke metrics (both qualitative and quantitative) to assess its interactions, outputs and outcomes, which are presented in a unique Research Embedment and Performance Profile, visualised in a radar chart. SIAMPI uses a mixed-methods case study approach to map three categories of productive interaction: direct personal contacts, indirect contacts such as publications, and financial or material links. These approaches have theoretical elegance, and some detailed empirical analyses were published as part of the SIAMPI final report [48]. However, neither approach has had significant uptake elsewhere in health research – perhaps because both are complex, resource-intensive and do not allow easy comparison across projects or programmes.

Whilst extending impact to include broader societal categories is appealing, the range of societal impacts described in different publications, and the weights assigned to them, vary widely; much depends on the researchers’ own subjective ratings. An attempt to capture societal impact (the Research Quality Framework) in Australia in the mid-2000s was planned but later abandoned following a change of government [49].

### UK Research Excellence Framework

The 2014 REF – an extensive exercise to assess UK universities’ research performance – allocated 20 % of the total score to research impact [50]. Each institution submitted an impact template describing its strategy and infrastructure for achieving impact, along with several four-page impact case studies, each of which described a programme of research, claimed impacts and supporting evidence. These narratives, which were required to follow a linear and time-bound structure (describing research undertaken between 1993 and 2013, followed by a description of impact occurring between 2008 and 2013) were peer-reviewed by an intersectoral assessment panel representing academia and research users (industry and policymakers) [50]. Other countries are looking to emulate the REF model [51].

An independent evaluation of the REF impact assessment process by RAND Europe (based on focus groups, interviews, survey and documentary analysis) concluded that panel members perceived it as fair and robust and valued the intersectoral discussions, though many felt the somewhat crude scoring system (in which most case studies were awarded 3, 3.5 or 4 points) lacked granularity [52]. The 6679 non-redacted impact case studies submitted to the REF (1594 in medically-related fields) were placed in the public domain (http://results.ref.ac.uk) and provide a unique dataset for further analysis.

In its review of the REF, the members of Main Panel A, which covered biomedical and health research, noted that “*International MPA* [Main Panel A] *members cautioned against attempts to ‘metricise’ the evaluation of the many superb and well-told narrations describing the evolution of basic discovery to health, economic and societal impact*” [50].

## Approaches with potential for the future

The approaches in this section, most of which have been recently developed, have not been widely tested but may hold promise for the future.

### Electronic databases

Research funders increasingly require principal investigators to provide an annual return of impact data on an online third-party database. In the UK, for example, Researchfish® (formerly MRC e-Val but now described as a ‘federated system’ with over 100 participating organisations) allows funders to connect outputs to awards, thereby allowing aggregation of all outputs and impacts from an entire funding stream. The software contains 11 categories: publications, collaborations, further funding, next destination (career progression), engagement activities, influence on policy and practice, research materials, intellectual property, development of products or interventions, impacts on the private sector, and awards and recognition.

Provided that researchers complete the annual return consistently and accurately, such databases may overcome some of the limitations of one-off, resource-intensive case study approaches. However, the design (and business model) of Researchfish® is such that the only funding streams captured are from organisations prepared to pay the membership fee, thereby potentially distorting the picture of whose input accounts for a research team’s outputs.

Researchfish® collects data both ‘top-down’ (from funders) and ‘bottom-up’ (from individual research teams). A comparable US model is the High Impacts Tracking System, a web-based software tool developed by the National Institute of Environmental Health Sciences; it imports data from existing National Institutes of Health databases of grant information as well as the texts of progress reports and notes of programme managers [53].

Whilst electronic databases are increasingly mainstreamed in national research policy (Researchfish® was used, for example, to populate the Framework on Economic Impacts described by the UK Department of Business, Innovation and Skills [54]), we were unable to identify any published independent evaluations of their use.

### Realist evaluation

**Fig. 3:**
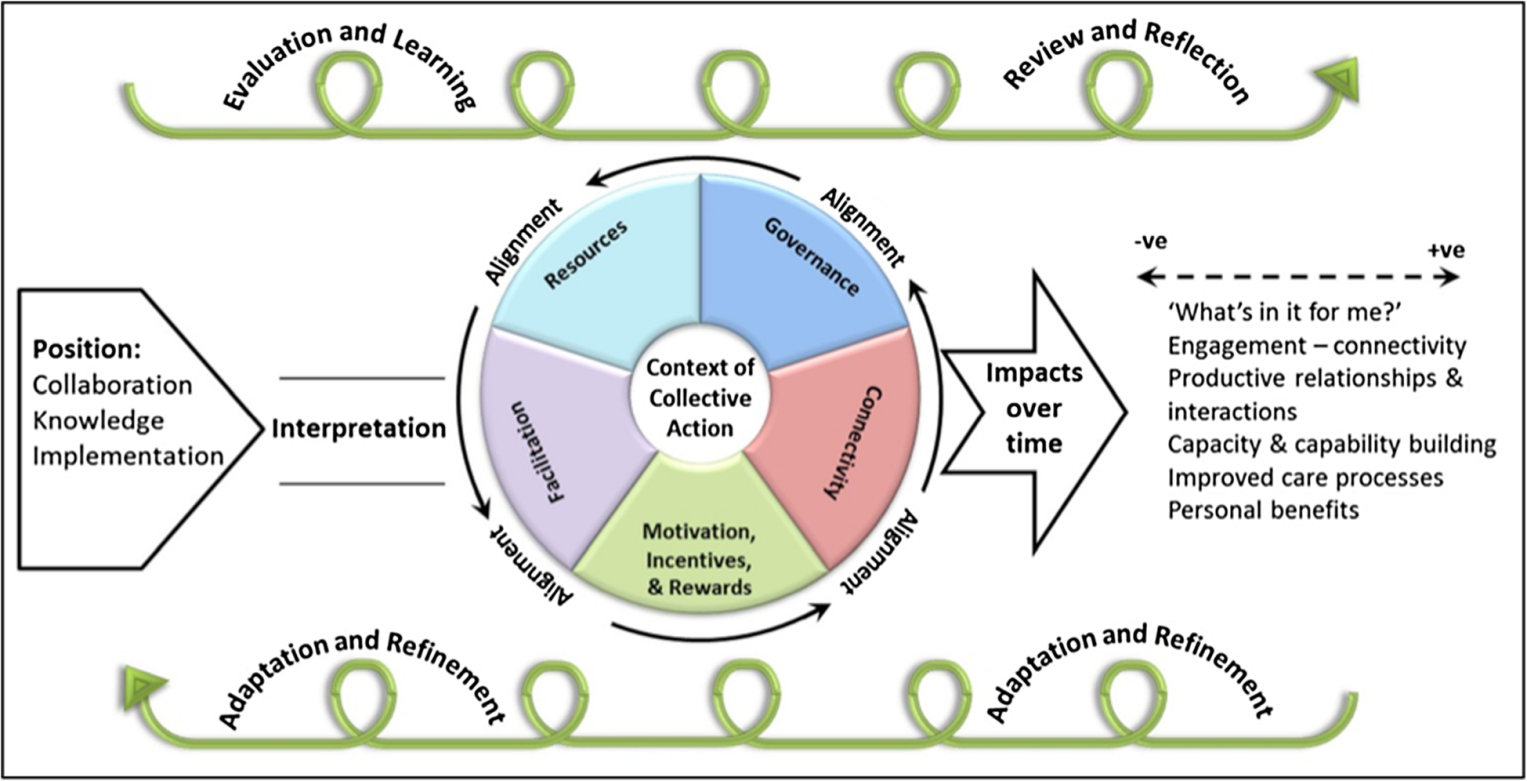
Realist model of research-service links and impacts in CLAHRCs (reproduced under UK non-commercial government licence from [56])

Realist evaluation, designed to address the question “what works for whom in what circumstances”, rests on the assumption that different research inputs and processes in different contexts may generate different outcomes (column 4 in Table 1) [55]. A new approach, developed to assess and summarise impact in the national evaluation of UK Collaborations for Leadership in Applied Health Research and Care, is shown in Fig. 3 [56]. Whilst considered useful in that evaluation, it was resource-intensive to apply.

### Contribution mapping

**Fig. 4:**
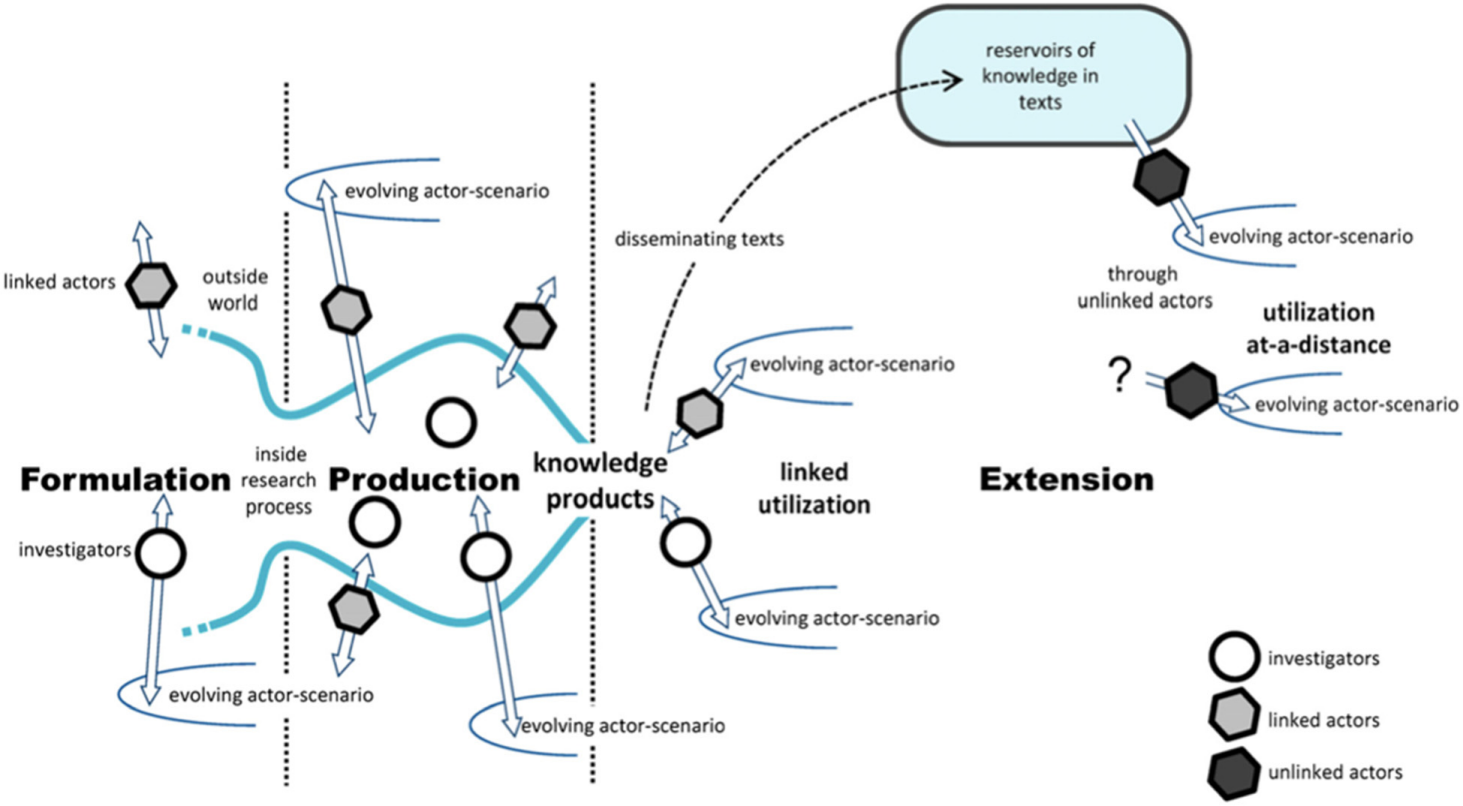
Kok and Schuit’s ‘contribution mapping’ model (reproduced under Creative Commons Attribution Licence 4.0 from [57])

Kok and Schuit describe the research ecosystem as a complex and unstable network of people and technologies [57]. They depict the achievement of impact as shifting and stabilising the network’s configuration by mobilising people and resources (including knowledge in material forms, such as guidelines or software) and enrolling them in changing ‘actor scenarios’. In this model, the focus is shifted from attribution to contribution – that is, on the activities and alignment efforts of different actors (linked to the research and, more distantly, unlinked to it) in the three phases of the research process (formulation, production and extension; Fig. 4). Contribution mapping, which can be thought of as a variation on the Dutch approaches to societal impact assessment described above, uses in-depth case study methods but differs from more mainstream approaches in its philosophical and theoretical basis (column 6 in Table 1), in its focus on processes and activities, and in its goal of producing an account of how the network of actors and artefacts shifts and stabilises (or not). Its empirical application to date has been limited.

### The SPIRIT Action Framework

**Fig. 5:**
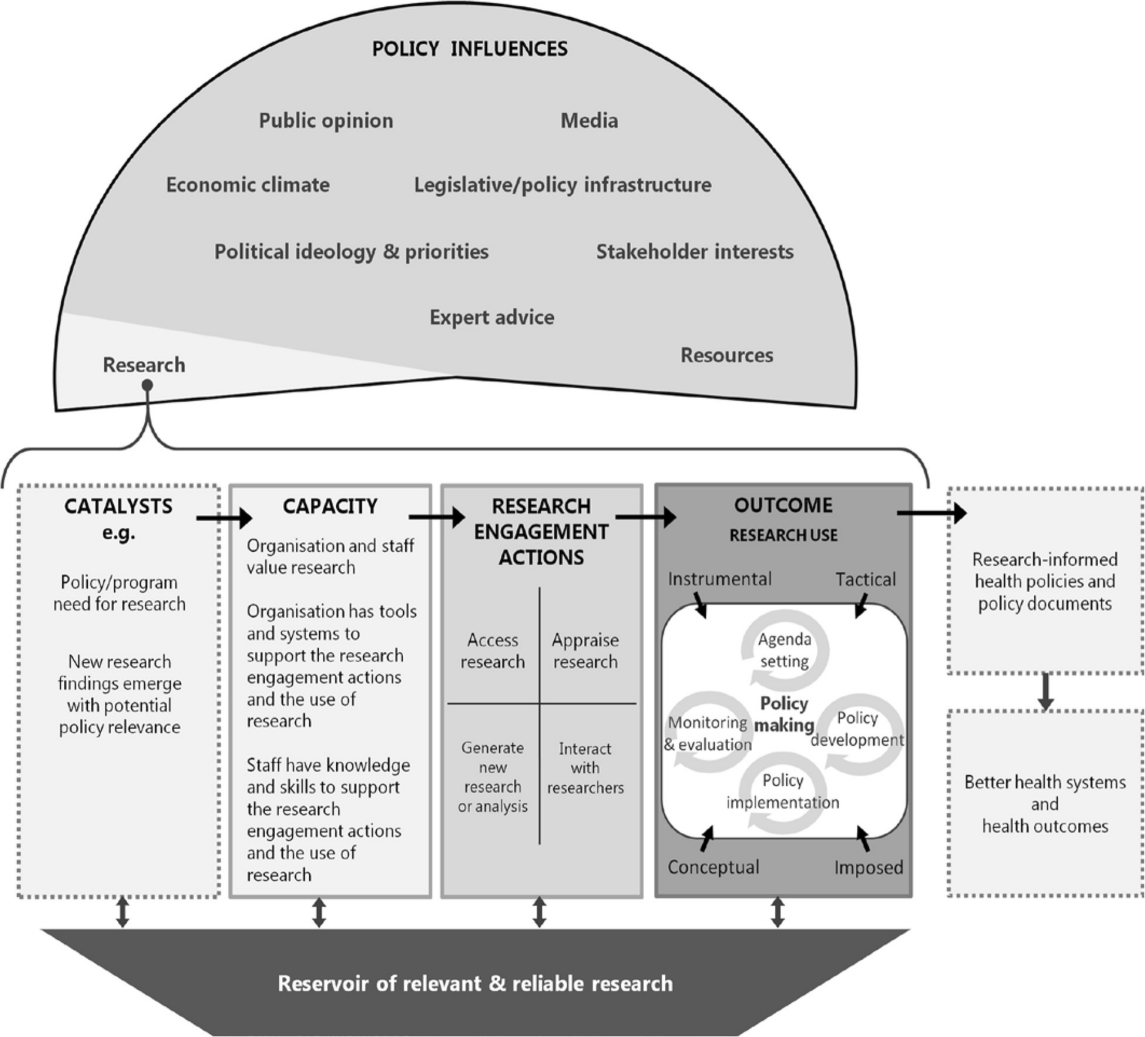
The SPIRIT Action Framework (reproduced under Creative Commons Attribution Licence from [58] Fig. 1, p. 151)

The SPIRIT Action Framework, recently published by Australia’s Sax Institute [58], retains a logic model structure but places more emphasis on engagement and capacity-building activities in organisations and acknowledges the messiness of, and multiple influences on, the policy process (Fig. 5). Unusually, the ‘logic model’ focuses not on the research but on the receiving organisation’s need for research. We understand that it is currently being empirically tested but evaluations have not yet been published.

### Participatory research impact model

Community-based participatory research is predicated on a critical philosophy that emphasises social justice and the value of knowledge in liberating the disadvantaged from oppression (column 5 in Table 1) [59]. Cacari-Stone et al.’s model depicts the complex and contingent relationship between a community-campus partnership and the policymaking process [60]. Research impact is depicted in synergistic terms as progressive strengthening of the partnership and its consequent ability to influence policy decisions. The paper introducing the model includes a detailed account of its application (Table 2), but beyond those, it has not yet been empirically tested.

## Discussion

This review of research impact assessment, which has sought to supplement rather than duplicate more extended overviews [1–7], prompts four main conclusions.

First, one size does not fit all. Different approaches to measuring research impact are designed for different purposes. Logic models can be very useful for tracking the impacts of a funding stream from award to quantitised (and perhaps monetised) impacts. However, when exploring less directly attributable aspects of the research-impact link, narrative accounts of how these links emerged and developed are invariably needed.

Second, the perfect is the enemy of the good. Producing detailed and validated case studies with a full assessment of context and all major claims independently verified, takes work and skill. There is a trade-off between the quality, completeness and timeliness of the data informing an impact assessment, on the one hand, and the cost and feasibility of generating such data on the other. It is no accident that some of the most theoretically elegant approaches to impact assessment have (ironically) had limited influence on the assessment of impact in practice.

Third, warnings from critics that focusing on short-term, proximal impacts (however accurately measured) could create a perverse incentive against more complex and/or politically sensitive research whose impacts are likely to be indirect and hard to measure [61–63] should be taken seriously. However, as the science of how to measure intervening processes and activities advances, it may be possible to use such metrics creatively to support and incentivise the development of complementary assets of various kinds.

Fourth, change is afoot. Driven by both technological advances and the mounting economic pressures on the research community, labour-intensive impact models that require manual assessment of documents, researcher interviews and a bespoke narrative may be overtaken in the future by more automated approaches. The potential for ‘big data’ linkage (for example, supplementing Researchfish® entries with bibliometrics on research citations) may be considerable, though its benefits are currently speculative (and the risks unknown).

## Conclusions

As the studies presented in this review illustrate, research on research impact is a rapidly growing interdisciplinary field, spanning evidence-based medicine (via sub-fields such as knowledge translation and implementation science), health services research, economics, informatics, sociology of science and higher education studies. One priority for research in this field is an assessment of how far the newer approaches that rely on regular updating of electronic databases are able to provide the breadth of understanding about the nature of the impacts, and how they arise, that can come for the more established and more ‘manual’ approaches. Future research should also address the topical question of whether research impact tools could be used to help target resources and reduce waste in research (for example, to decide whether to commission a new clinical trial or a meta-analysis of existing trials); we note, for example, the efforts of the UK National Institute for Health Research in this regard [64].

Once methods for assessing research impact have been developed, it is likely that they will be used. As the range of approaches grows, the challenge is to ensure that the most appropriate one is selected for each of the many different circumstances in which (and the different purposes for which) people may seek to measure impact. It is also worth noting that existing empirical studies have been undertaken primarily in high-income countries and relate to health research systems in North America, Europe and Australasia. The extent to which these frameworks are transferable to low- or middle-income countries or to the Asian setting should be explored further.

## Box 1: Definitions of research impact

Impact is the effect research has beyond academia and consists of “*….benefits to one or more areas of the economy, society, culture, public policy and services, health, production, environment, international development or quality of life, whether locally, regionally, nationally or internationally*” (paragraph 62) and as “*…manifested in a wide variety of ways including, but not limited to: the many types of beneficiary (individuals, organisations, communities, regions and other entities); impacts on products, processes, behaviours, policies, practices; and avoidance of harm or the waste of resources.*” (paragraph 63)

UK 2014 Research Excellence Framework [65]

“*‘Health impacts’ can be defined as changes in the healthy functioning of individuals (physical, psychological, and social aspects of their health), changes to health services, or changes to the broader determinants of health. ‘Social impacts’ are changes that are broader than simply those to health noted above, and include changes to working systems, ethical understanding of health interventions, or population interactions. ‘Economic impacts’ can be regarded as the benefits from commercialization, the net monetary value of improved health, and the benefits from performing health research.*”

Canadian Academy of Health Sciences [33] (p. 51)

Academic impact is “*The demonstrable contribution that excellent research makes to academic advances, across and within disciplines, including significant advances in understanding, methods, theory and application.*” Economic and societal impact is “*fostering global economic performance, and specifically the economic competitiveness of the UK*, *increasing the effectiveness of public services and policy,* [and] *enhancing quality of life, health and creative output.*”

Research Councils UK Pathways to Impact (http://www.rcuk.ac.uk/innovation/impacts/)

“*A research impact is a recorded or otherwise auditable occasion of influence from academic research on another actor or organization.* […] *It is not the same thing as a change in outputs or activities as a result of that influence, still less a change in social outcomes. Changes in organizational outputs and social outcomes are always attributable to multiple forces and influences. Consequently, verified causal links from one author or piece of work to output changes or to social outcomes cannot realistically be made or measured in the current state of knowledge.* […] *However, secondary impacts from research can sometimes be traced at a much more aggregate level, and some macro-evaluations of the economic net benefits of university research are feasible. Improving our knowledge of primary impacts as occasions of influence is the best route to expanding what can be achieved here.*”

London School of Economics Impact Handbook for Social Scientists [66]
